# Retrospective analysis of feline intestinal parasites: trends in testing positivity by age, USA geographical region and reason for veterinary visit

**DOI:** 10.1186/s13071-020-04319-4

**Published:** 2020-09-15

**Authors:** Sarah Sweet, Donald Szlosek, Donald McCrann, Michael Coyne, David Kincaid, Evan Hegarty

**Affiliations:** IDEXX Laboratories, Inc., One IDEXX Drive, Westbrook, ME USA

**Keywords:** Cat, Feline, Parasitism, Fecal, Flotation, Coproantigen, Hookworm, Whipworm, Ascarid, *Giardia*

## Abstract

**Background:**

The goals of this retrospective study were to estimate parasite positivity in samples from cats using zinc sulfate fecal flotation by centrifugation (“centrifugation”) and coproantigen and examine trends with age, geographical region and reason for visit to veterinarian. Common methods of parasite detection, such as centrifugal flotation, passive flotation, or direct smear, may underrepresent the true prevalence of intestinal parasites in cats. Coproantigen testing detects more positive samples than traditional methods alone.

**Methods:**

Feline fecal test results from the continental USA containing results for fecal exams performed using centrifugation paired with coproantigen results for ascarid, hookworm, whipworm and *Giardia* were obtained from the database of a national commercial reference laboratory comprised of multiple regional sites.

**Results:**

Parasite positivity was highest in samples from young cats and decreased with cat age. The western region of the USA had lower total parasite positivity than other regions for all parasites except *Giardia*. Cats receiving fecal tests during veterinary wellness visits had only slightly lower parasite positivity than samples from cats during sick clinical visits.

**Conclusions:**

This study showed a larger population of cats are at increased risk of parasitism than commonly believed and coproantigen testing produces more positive test results for the four parasites that antigen can detect than centrifugation of feline fecal samples.
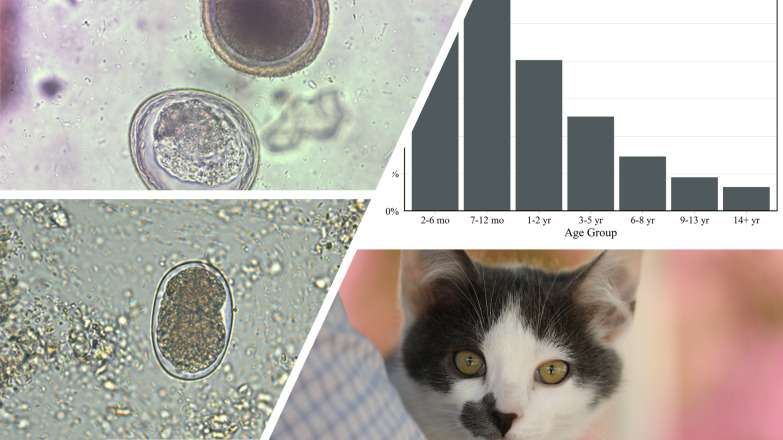

## Background

Intestinal parasite risk to pet cats is underrepresented in the literature and is often overlooked by pet owners and veterinarians [1–3]. The aims of this study were to report the proportion of positive fecal test results in samples from pet cats in the United States; to examine trends in positivity with age, geographical region, and reason for veterinary visit; and to describe differences in positivity between the two diagnostic methods, centrifugation and coproantigen immunoassay. Previously published studies investigating feline intestinal parasitism in the USA were limited to a single parasite [1, 4, 5], a particular geographical region [2, 3, 6], or focused solely on free-roaming or shelter cats [2, 3, 7]. These limitations make it difficult for a veterinarian to assess the risk to their patients.

Reported prevalence of intestinal parasite infection in domestic cats is often region-specific and may vary widely, ranging from 0.03 to 33% [8–12]. One resource for national reporting on feline fecal parasitism is CAPC (Companion Animal Parasite Council) [13] which sources its data from fecal submissions to commercial veterinary reference laboratories (IDEXX Reference Laboratories, Inc., Westbrook, ME, USA, Antech Diagnostics and Imaging, Fountain Valley, CA USA). While CAPC prevalence maps provide local, state and regional data, they ignore age and health status of the patient.

Fecal flotation is the most commonly used method of parasite identification in veterinary medicine. This technique may underrepresent prevalence due to prepatent or single-sex infections [14] and when conducted in clinic, result validity is highly dependent on sample preparation, type and concentration of flotation solution and reader skill [15, 16]. Coproantigen tests have previously been shown to increase the ability to detect infections [12]. The addition of coproantigen immunoassay detection to fecal diagnostics should increase the detection of feline intestinal parasite infections in the USA.

## Methods

Results ordered by veterinary practices in the USA were collected from samples submitted to a national commercial reference laboratory corporation using proprietary software (IDEXX Laboratories, Inc. Westbrook, ME, USA) from January 2017 to December 2018. A single centrifugation for 5 min at 500× *g* was performed using zinc sulfate flotation solution (specific gravity 1.24) then read at 100× and 400× magnifications following standard methods [17]. Coproantigen immunoassays utilized unique capture and detection antibodies developed against recombinantly expressed proteins of ascarids (*Toxocara* spp., *Toxascaris* spp., *Baylisascaris* spp.), hookworms (*Ancylostoma* spp., *Uncinaria* spp.) and whipworms (*Trichuris* spp.) and *Giardia* spp. [17–19]. Presence of *Cystoisospora* spp., lungworms (*Filaroides* spp., *Aelurostrongylus* spp., *Angiostrongylus* spp., *Eucoleus* spp., *Capillaria* spp.), tapeworms (cestodes) (*Dipylidium* spp., *Moniezia* spp., *Anoplocephala* spp., *Spirometra* spp., *Taenia* spp., *Mesocestoides* spp., *Diphyllobothrium* spp., *Echinococcus* spp., *Hymenolepis* spp., *Paranoplocephala* spp., *Cittotaenia* spp.) and trematodes (*Paragonimus* spp., *Alaria* spp., *Nanophyetus* spp., *Platynosomum* spp.), was only detected *via* centrifugation and these parasites are reported as well.

Samples from cats two months to 25 years of age, tested for hookworm, ascarid, whipworm and *Giardia* spp. with centrifugation and coproantigen (IDEXX Laboratories, Inc. Westbrook, ME, USA) on the same visit were used in the study. To examine if there was bias in submission, e.g. samples suspected of being positive were more likely to be submitted for both tests, centrifugation positivity by parasite for these groups was compared. While centrifugation positive percentages tended to be higher in samples submitted for testing by both methods, the differences did not exceed 0.5 percentage points for any parasite.

For inclusion in the study, age and geographical location of the clinic that provided the sample were required. For cats with multiple samples within the dataset, only the first sample for an individual cat was included in the analysis. Only samples from clinical visits were included; samples from visits that were classified as retail, grooming, or boarding were excluded. Clinical visits were subdivided into wellness and other clinical visits. Wellness visits were visits to the veterinarian for annual exams, vaccinations, or routine check-ups. Other clinical visits were those for a new illness, monitoring an existing illness, or for a procedure such as surgery or teeth cleaning.

All data analyses were conducted using R version 3.5.3 [20]. Data analysis was done with the tidyverse and multiple helper functions within the following packages: *data.table* [21], *magrittr* [22], *here* [23], *Hmisc* [24], *ggplot2* [25], *gridExtra* [26], *ggpubr* [27] and *extrafont* [28]. Summary statistics were reported as the percent positive of all test results. Confidence intervals were calculated using the binomial exact method. Significant differences in percent positive among subgroups of samples were determined by non-overlapping confidence intervals. For regional analysis of parasite positivity, United States Census Bureau regions were used, as these align with major geographical and demographical differences that could theoretically influence parasite prevalence [29].

## Results

**Fig. 1:**
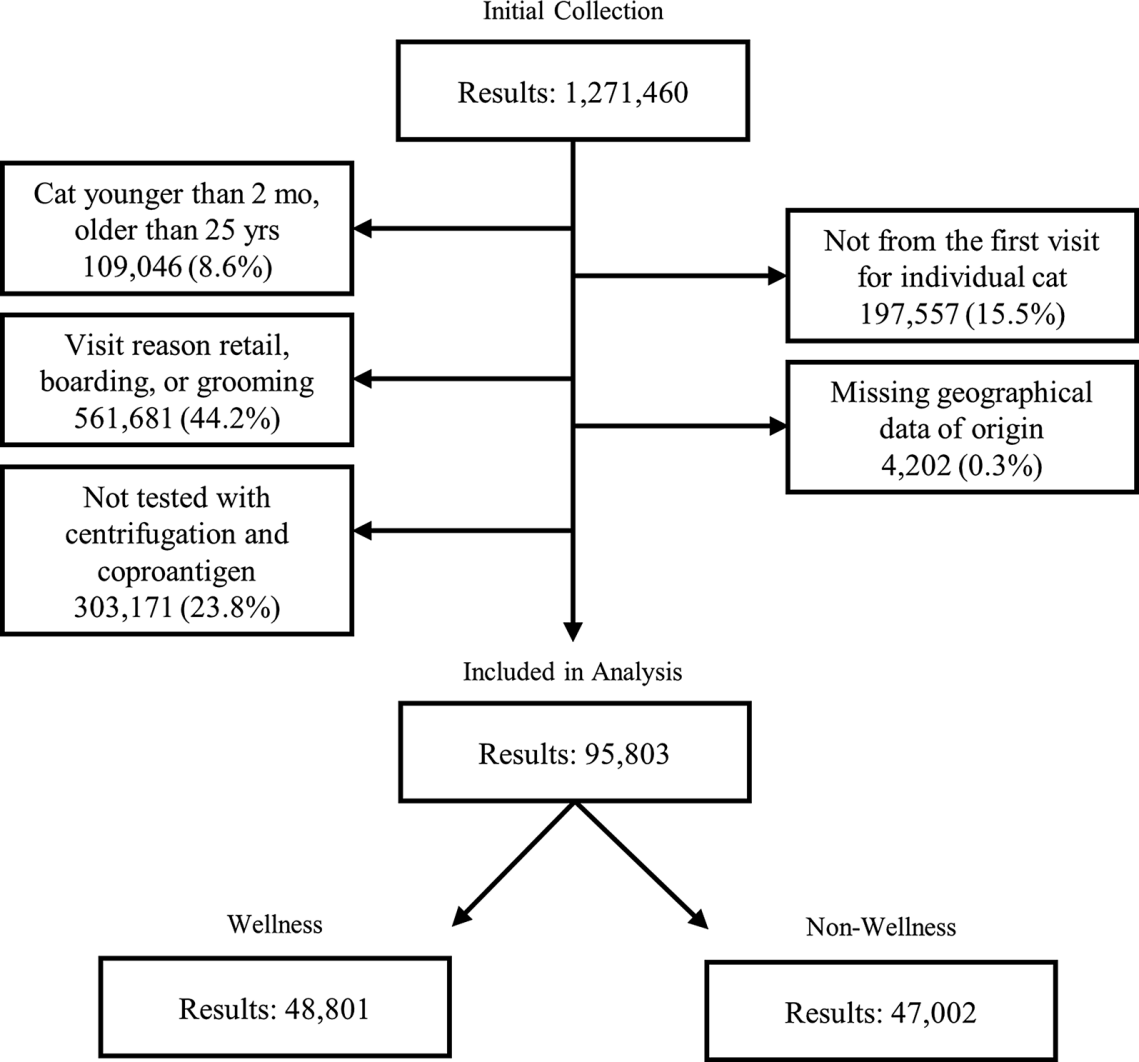
Consort diagram of the exclusion criteria and study population

A total of 1,271,460 feline fecal test results were obtained. Fecal test results from cats younger than two months and older than 25 years of age (*n* = 109,046) and any subsequent test results on cats already in the study (*n* = 197,557) were excluded. Sample results obtained from cats during non-clinical visits (*n* = 561,681), with missing geographical data (*n* = 4202), or not tested with both coproantigen and centrifugation (*n* = 303,171) were also excluded. The study population was comprised of fecal test results from 95,803 cats, with 51.0% (*n* = 48,801) obtained during wellness visits and 49.0% (*n* = 47,002) obtained during other clinical visits (Fig. 1). Approximately, 1.4% of the test results had more than one parasite identified. The highest co-infection was ascarid + *Giardia* which occurred in 0.9% of all fecal tests. Additional co-infection information can be found in Additional file 1: Table S1.

**Fig. 2:**
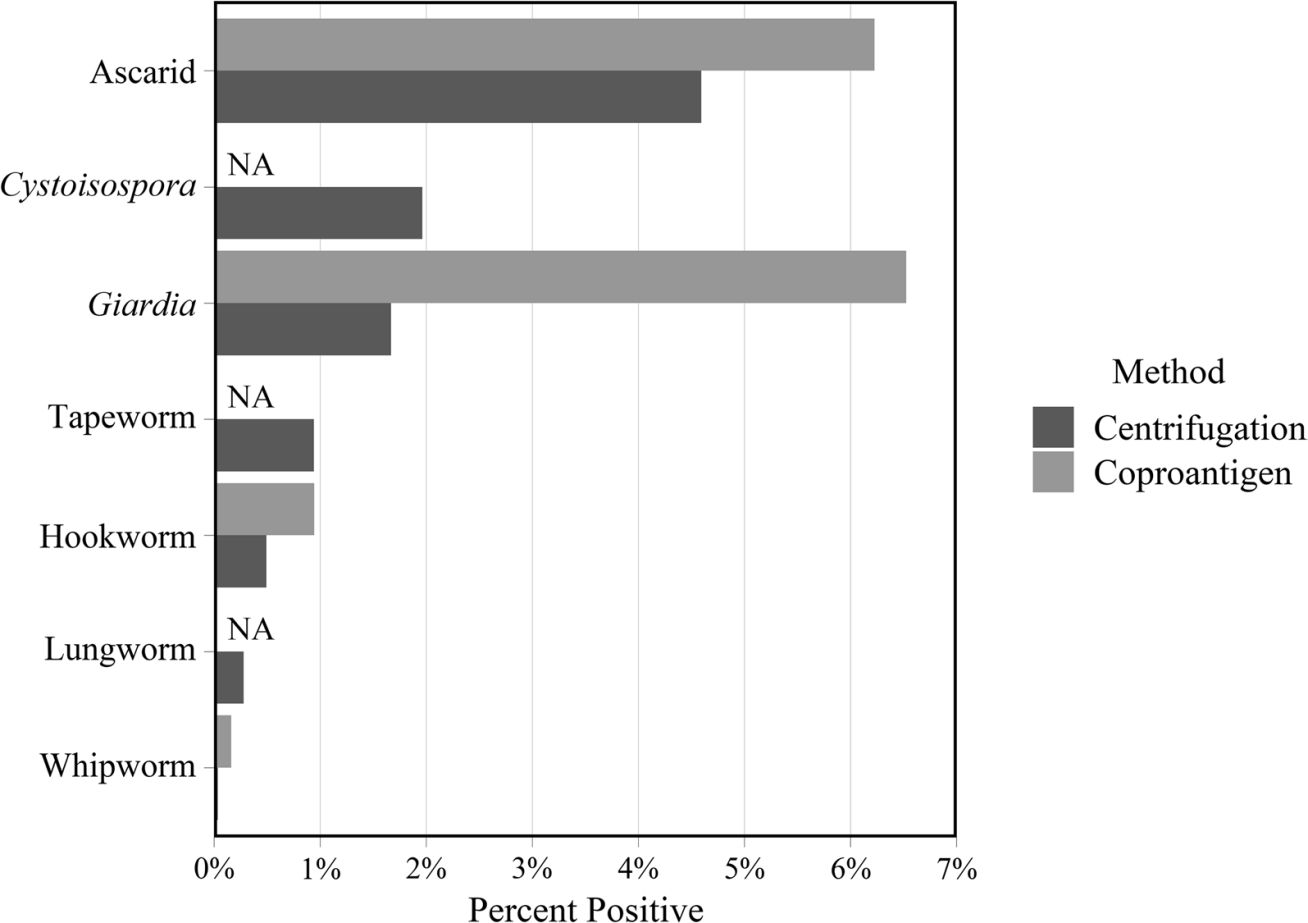
Proportion of cats with a positive test result by centrifugation or coproantigen

In the study population, ascarids were the most common parasites identified by centrifugation, with a positivity of 4.6%, followed by *Cystoisospora* (2%), *Giardia* (1.7%), tapeworms (0.9%), hookworms (0.5%), lungworms (0.3%) and whipworms (0.03%) (Fig. 2, Table 1). Ascarids and *Giardia* had the highest percent positive on coproantigen with 6.2% and 6.5% positive results, respectively. For each of the intestinal parasites tested by both diagnostic methods, the proportion of positive tests was significantly higher by coproantigen than by centrifugation (Table 1).

**Table 1:** Positive test results for feline intestinal parasites by centrifugation and coproantigen by age

Parasite	Method	2–6 months (%) (95% CI)	7–12 months (%) (95% CI)	1–2 years (%) (95% CI)	3–5 years (%) (95% CI)	6–8 years (%) (95% CI)	9–13 years (%) (95% CI)	14+ years (%) (95% CI)	Total (%) (95% CI)
Ascarid	CE	12.4 (12.0–2.9)	8.1 (7.4–8.8)	4.8 (4.3–5.3)	2.9 (2.6–3.2)	1.7 (1.5–1.9)	1.0 (0.9–1.2)	0.6 (0.5–0.8)	4.6 (4.5–4.7)
	CO	16.5 (16.0–17.0)	9.8 (9.1–10.5)	6.2 (5.7–6.8)	4.2 (3.9–4.5)	2.5 (2.2–2.8)	1.6 (1.5–1.8)	1.2 (1.0–1.4)	6.2 (6.1–6.4)
*Cystoisospora*	CE	6.3 (6.0–6.7)	2.3 (2.0–2.7)	1.5 (1.3–1.8)	0.8 (0.7–0.9)	0.6 (0.5–0.7)	0.4 (0.3–0.5)	0.3 (0.2–0.4)	2.0 (1.9–2.1)
*Giardia*	CE	3.2 (3.0–3.5)	4.8 (4.4–5.4)	2.6 (2.3–3.0)	1.5 (1.3–1.7)	0.8 (0.6–0.9)	0.2 (0.2–0.3)	0.2 (0.1–0.3)	1.7 (1.6–1.8)
	CO	12.4 (11.9–12.8)	16.6 (15.7–17.5)	11.0 (10.4–11.7)	6.1 (5.7–6.5)	2.8 (2.5–3.1)	1.4 (1.3–1.6)	1.0 (0.9–1.2)	6.5 (6.4–6.7)
Tapeworm	CE	1.1 (1.0–1.3)	1.5 (1.3–1.9)	1.6 (1.4–1.9)	1.4 (1.2–1.6)	0.8 (0.7–1.0)	0.5 (0.4–0.6)	0.2 (0.1–0.3)	0.9 (0.9–1.0)
Hookworm	CE	0.5 (0.4–0.6)	1.4 (1.1–1.7)	1.1 (0.9–1.4)	0.6 (0.5–0.7)	0.3 (0.2–0.4)	0.2 (0.2–0.3)	0.1 (0.1–0.2)	0.5 (0.5–0.5)
	CO	1.2 (1.1–1.4)	1.8 (1.5–2.2)	1.8 (1.6–2.1)	1.0 (0.8–1.2)	0.6 (0.5–0.8)	0.4 (0.4–0.5)	0.4 (0.3–0.6)	0.9 (0.9–1.0)
Lungworm	CE	0.6 (0.5–0.8)	0.5 (0.3–0.7)	0.5 (0.3–0.6)	0.2 (0.1–0.3)	0.1 (0.1–0.2)	0.1 (0.0–0.1)	0.1 (0.0–0.1)	0.3 (0.2–0.3)
Whipworm	CE	0.1 (0.0–0.1)	0.1 (0.0–0.2)	0.1 (0.0–0.2)	0.0 (0.0–0.1)	0.0 (0.0–0.0)	0.0 (0.0–0.0)	0.0 (0.0–0.0)	0.0 (0.0–0.0)
	CO	0.2 (0.1–0.3)	0.2 (0.1–0.4)	0.2 (0.1–0.3)	0.2 (0.1–0.3)	0.2 (0.1–0.2)	0.1 (0.1–0.2)	0.1 (0.0–0.1)	0.2 (0.1–0.2)

*Abbreviations*: CE, centrifugation; CO, coproantigen

Most fecal samples that were positive for a parasite by centrifugation also tested positive by coproantigen for the same parasite, excepting whipworms, which had slightly less than half of the positives by centrifugation also positive by coproantigen. Of the fecal samples that tested positive for ascarids by either method, 7.7% were positive by centrifugation alone, 32.0% were positive by coproantigen alone and the remaining 60.3% were positive by both methods. For *Giardia*, the positivity by centrifugation alone was 1%, 74.7% by antigen alone and 24.3% by both. For hookworms, the positivity by centrifugation alone was 17.4%, 56.8% by antigen alone and 25.8% by both. For whipworms, the positivity by centrifugation alone was 10.4%, 80.4% by antigen alone and 9.2% by both. Numbers of positive tests by each method are detailed in Additional file 1: Table S1.

**Fig. 3:**
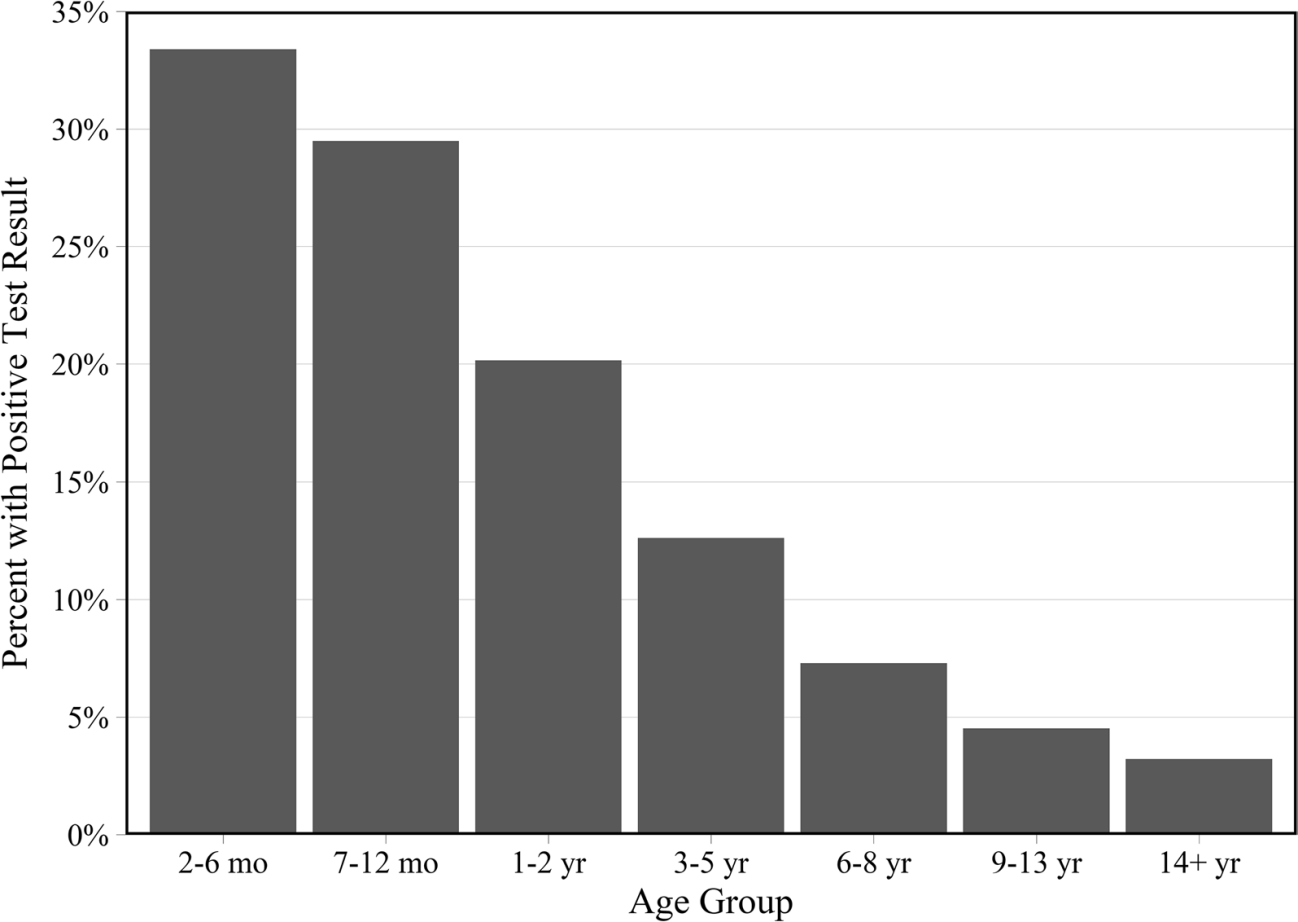
Proportion of cats with a positive test result by either centrifugation or coproantigen by age

**Fig. 4:**
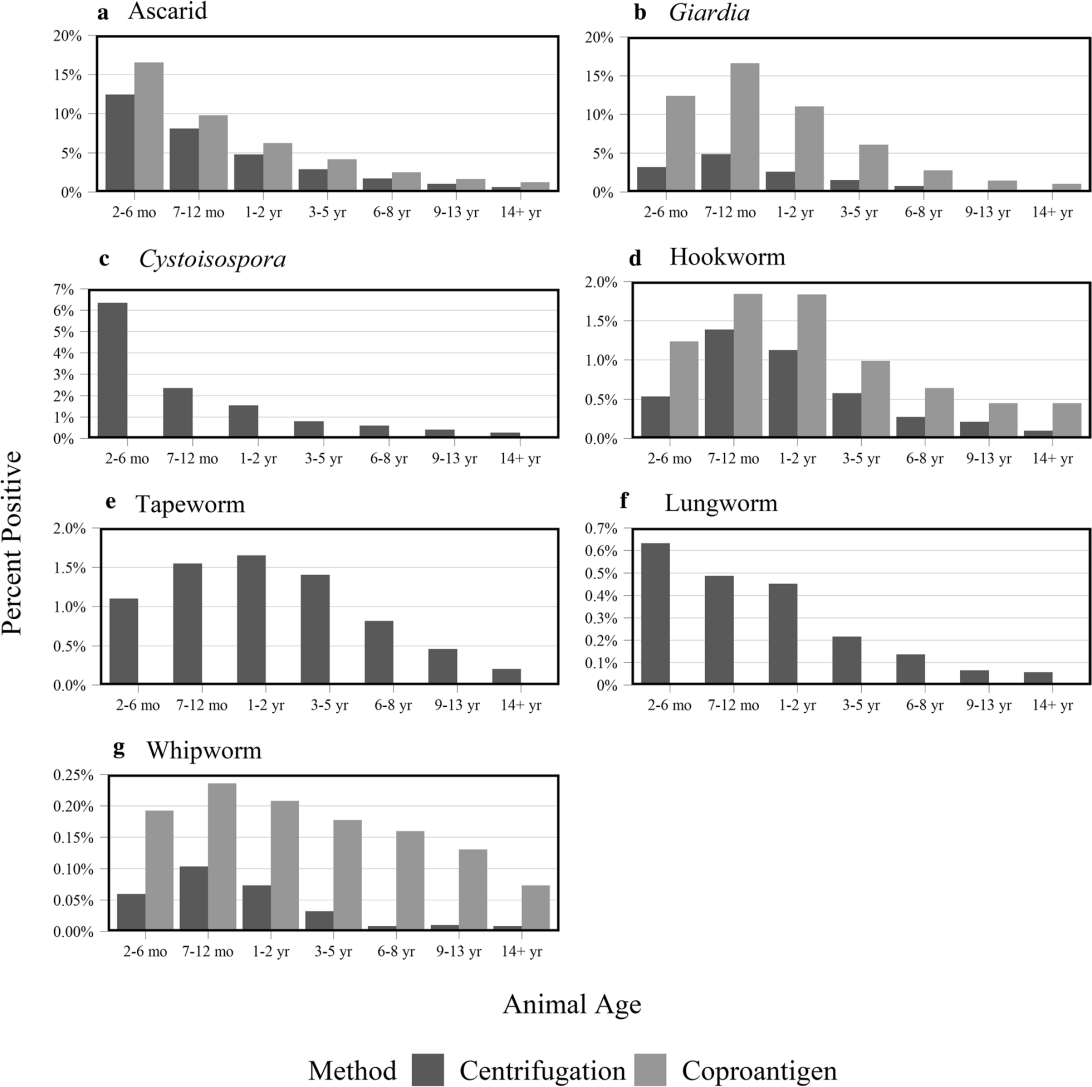
Proportion of cats with positive test results by centrifugation and coproantigen by age. **a** Ascarid. **b** *Giardia*. **c** *Cystoisospora*. **d** Hookworm. **e** Tapeworm. **f** Lungworm. **g** Whipworm

A general trend of decreasing positive proportions of test results was observed as the age of the cat increases (Fig. 3). However, examining the age trend in positivity by parasite showed that for *Giardia*, hookworms, tapeworms and whipworms, the highest proportion of positive results was on samples from cats either 7–12 months, or 1–2 years of age (Fig. 4, Table 1). These trends are consistent between centrifugation and coproantigen methods, where applicable. The ages of cats receiving centrifugation or coproantigen tests on wellness or non-wellness veterinary visits can be found in Additional file 2: Table S2.

**Fig. 5:**
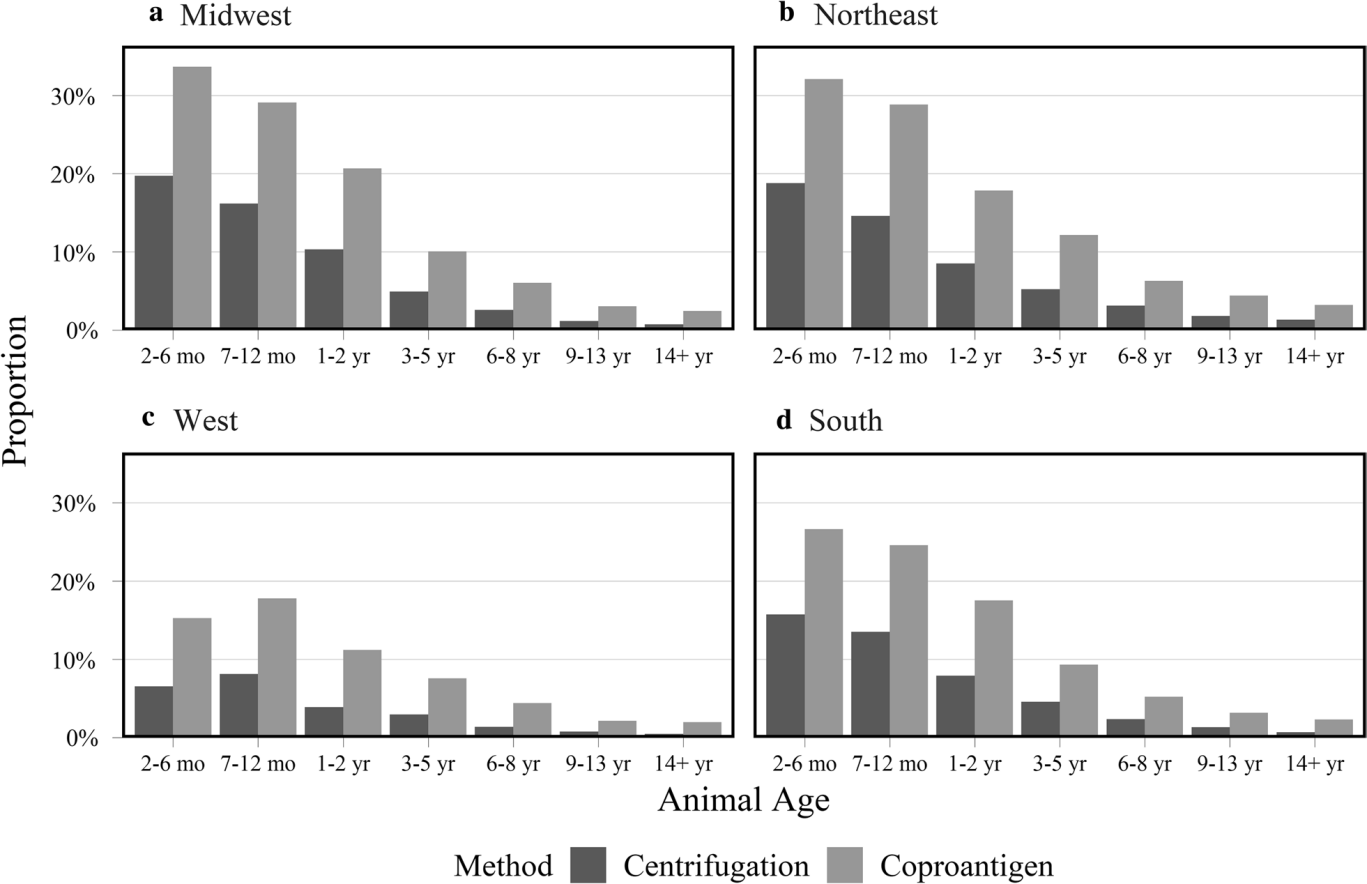
Proportion of test results positive for ascarid, *Giardia*, hookworm, or whipworm by centrifugation and coproantigen by United States Census Bureau regions and age category. **a** Midwest. **b** Northeast. **c** West. **d** South

**Fig. 6:**
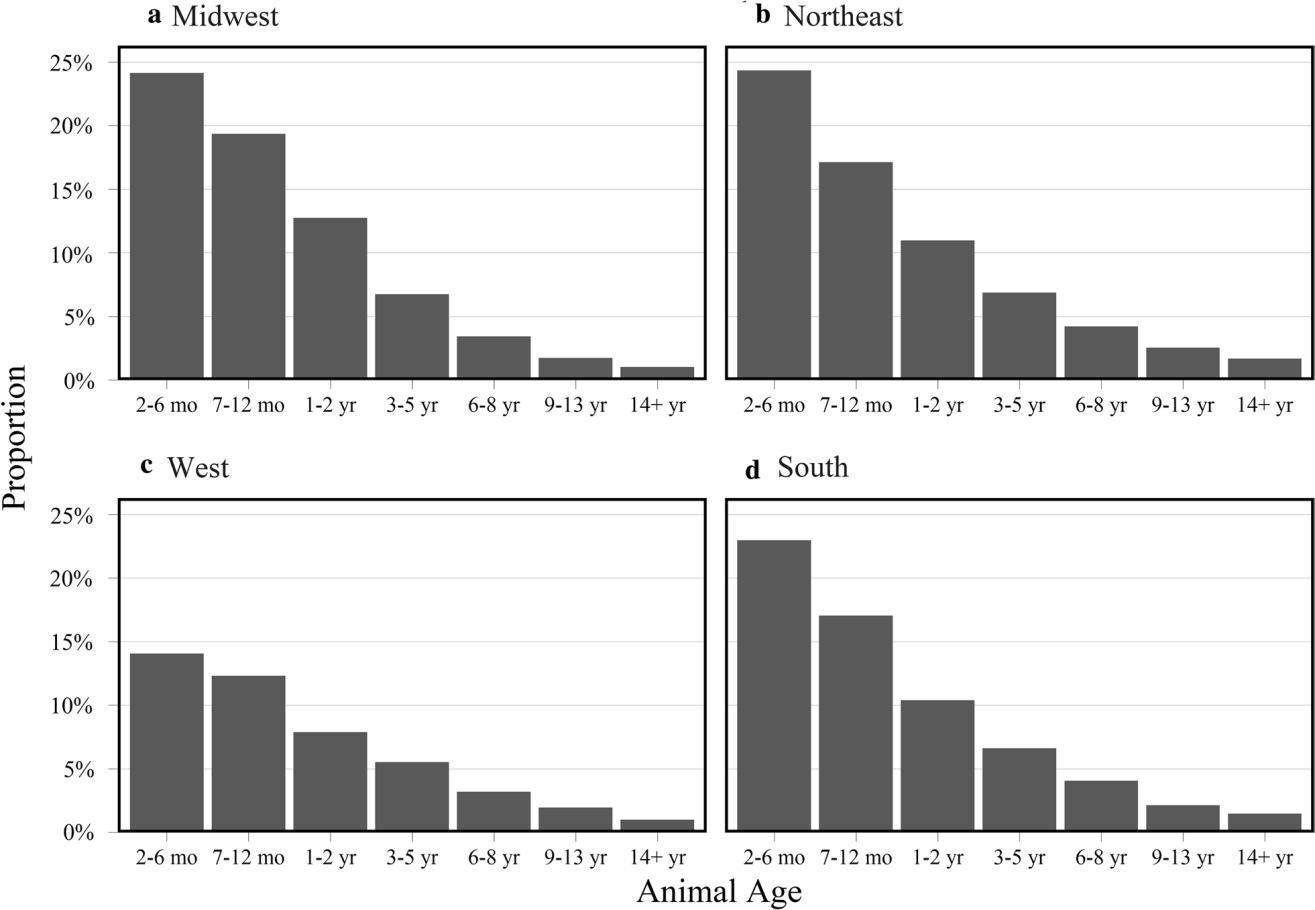
Proportion of test results positive for ascarid, *Giardia*, hookworm, or whipworm by either centrifugation or coproantigen by United States Census Bureau regions and age category. **a** Midwest. **b** Northeast. **c** West. **d** South

Combined parasite test result positivity varied by region (including ascarids, *Giardia*, hookworms and whipworms). The lowest overall parasite positivity was found in samples from the western region, though *Giardia* positivity in this region was no less frequent than in other regions. The highest proportions of ascarid-positive samples were from the Northeast and the Midwest (Table 2). Whipworm and hookworm infections were the highest in samples from the South. Both *Cystoisospora* and tapeworms were highest in samples from southern and western regions (Table 2). When examined by age, category and region, samples from younger cats tended to have higher rates of positivity for hookworms, ascarids and *Giardia* in every region with more positive results found by coproantigen (Fig. 5, Additional file 3: Figure S1). In addition, this trend was also seen when centrifugation results from all parasites are combined (Fig. 6).

**Table 2:** Proportion of positive test results across all parasites by United States Census Bureau regions

Parasite	Midwest		Northeast		South		West	
	Centrifugation (%) (95% CI)	Coproantigen (%) (95% CI)	Centrifugation (%) (95% CI)	Coproantigen (%) (95% CI)	Centrifugation (%) (95% CI)	Coproantigen (%) (95% CI)	Centrifugation (%) (95% CI)	Coproantigen (%) (95% CI)
Ascarid	6.2 (5.8–6.5)	8.2 (7.8–8.6)	5.8 (5.6–6.1)	8.0 (7.7–8.2)	3.9 (3.6–4.2)	5.2 (4.9–5.6)	1.4 (1.2–1.6)	2.0 (1.8–2.2)
*Cystoisospora*	1.8 (1.6–2.0)	–	1.8 (1.7–1.9)	–	2.2 (1.9–2.4)	–	2.2 (2.0–2.5)	–
*Giardia*	2.0 (1.8–2.2)	7.5 (7.1–7.9)	1.4 (1.3–1.5)	6.6 (6.4–6.9)	1.7 (1.5–1.8)	5.9 (5.5–6.2)	2.0 (1.8–2.2)	6.1 (5.8–6.4)
Tapeworm	0.7 (0.6–0.8)	–	0.7 (0.6–0.8)	–	1.1 (1.0–1.3)	–	1.5 (1.3–1.7)	–
Hookworm	0.5 (0.4–0.6)	1.0 (0.8–1.1)	0.4 (0.4–0.5)	0.9 (0.8–1.0)	1.1 (0.9–1.3)	1.7 (1.5–1.9)	0.1 (< 0.1–0.1)	0.4 (0.3–0.5)
Lungworm	0.3 (0.3–0.4)	–	0.4 (0.4–0.5)	–	0.1 (0.1–0.2)	–	< 0.1 (< 0.1–0.1)	–
Whipworm	< 0.1(< 0.1–0.1)	0.2 (0.1–0.2)	< 0.1 (< 0.1–< 0.1)	0.1 (0.1–0.2)	0.1 (0.1–0.2)	0.3 (0.2–0.4)	< 0.1 (< 0.1–< 0.1)	0.1 (0.1–0.2)

In sample results from cats presenting for a wellness exam, ascarids had the highest positivity by centrifugation (5.3%), followed by *Cystoisospora* (2.2%), *Giardia* (1.5%), tapeworms (0.8%), hookworms (0.4%), lungworms (0.2%) and whipworms (<0.1%) (Table 3). No trematodes were identified in any cat sample in this study. Similarly, in sample results from cats presenting for wellness exams, ascarids also had the highest positivity by coproantigen (7.2%), followed by *Giardia* (6.2%), hookworms (0.9%) and whipworms (0.1%) (Table 3). While parasite positivity was significantly different for most parasites on wellness compared to non-wellness clinical visits, the differences were not clinically meaningful. Ascarids and *Cystoisospora* were found more often on wellness exams. Age-group differences by clinical exam type were observed. Kittens 2–6-months of age comprised 28% (*n* = 13,680) of the wellness exam population and only 14% (*n* = 6578) of the other clinical population (Additional file 4: Table S3). These age-group differences account for higher ascarid and *Cystoisospora* test positivity in wellness exams than other clinical visits, as kittens less than 6 months of age were disproportionately represented in wellness visits and have the highest rates of ascarid infections of any age group.

**Table 3:** Feline intestinal parasites by centrifugation or coproantigen on wellness or non-wellness veterinary visits

Parasite	Classification	Centrifugation (%) (95% CI)	Coproantigen (%) (95% CI)
Ascarid	Other clinical visit	3.9 (3.7–4.1)	5.2 (5.0–5.4)
	Wellness	5.3 (5.1–5.5)	7.2 (7.0–7.4)
*Cystoisospora*	Other clinical visit	1.7 (1.6–1.9)	–
	Wellness	2.2 (2.1–2.3)	–
*Giardia*	Other clinical visit	1.8 (1.7–2.0)	6.9 (6.7–7.1)
	Wellness	1.5 (1.4–1.6)	6.2 (6.0–6.4)
Tapeworm	Other clinical visit	1.1 (1.0–1.2)	–
	Wellness	0.8 (0.7–0.9)	–
Hookworm	Other clinical visit	0.6 (0.5–0.6)	1.0 (0.9–1.1)
	Wellness	0.4 (0.4–0.5)	0.9 (0.8–1.0)
Lungworm	Other clinical visit	0.3 (0.3–0.4)	–
	Wellness	0.2 (0.2–0.3)	–
Whipworm	Other clinical visit	< 0.1 (< 0.1–0.1)	0.2 (0.2–0.2)
	Wellness	< 0.1 (< 0.1–0.1)	0.1 (0.1–0.2)

## Discussion

The total parasite positivity rate in this study was largely driven by ascarid and *Giardia* test results regardless of method used. In addition, this study observed an age-dependent relationship, with fewer positive test results seen in samples from adult cats as compared to those from cats under two years of age. Ascarid infections were highest in samples from 2–6-month-old kittens, likely owing to absence of early deworming protocols and vertical transmission from queen to offspring [30, 31].

Coproantigen testing detected more positive samples for hookworms, ascarids, whipworms and *Giardia* than centrifugation. Coproantigen testing for nematode parasites uses ELISA immunoassay technology to detect antigens secreted or excreted by the parasite of interest. These antigens are unique to the mature, metabolically active parasites and do not cross-react with eggs [16–18]. *Giardia* spp. coproantigen testing detects a soluble cell wall antigen that is produced when the trophozoite encysts in the intestinal wall of the host [32]. Possible contributing factors to the difference in positive results may be difficulty identifying cysts or trophozoites *via* centrifugation, as with *Giardia* spp., or samples collected during a non-egg-shedding stage of the parasite’s life-cycle. Combined positive results for hookworms, ascarids, whipworms and *Giardia* using coproantigen showed similar trends among samples from all age groups and regions with a consistently higher number of positive results than centrifugation.

The most notable regional difference in proportion of positive tests was in the western USA, which had a lower proportion of positives than all other regions detected using either centrifugation or coproantigen testing. Proportion of positive samples declined with age in each region, with possible deviations from this age-related positivity trend seen with whipworms. Samples from the West had a spike in overall parasite positivity in the 7–12-month age range, inconsistent with trends in the other regions. This is because ascarid infections decreased in positivity with age of the cat and comprised most of the positive results in the South, the Northeast and the Midwest, whereas *Giardia* was the most common source of positive results in the West. The highest rate of *Giardia* infections was in samples from 7–12-month-old cats, causing this spike in test positivity in the West.

Coproantigen-only positive results were observed more frequently than positive results for centrifugation-only. Regardless, the proportion of samples from cats that tested positive was observed to decrease with age in both coproantigen and centrifugation across all regions and parasites. This similarity in trends of test-positivity suggests that both test methods are representative of the underlying prevalence in these populations. While these data report higher levels of test positivity for coproantigen testing than centrifugation, the true disease state is not known and whether these results represent a true infection, cannot be determined from these data. It should be noted that these comparisons were between coproantigen testing and fecal flotation with centrifugation as opposed to the more commonly used in-clinic flotation, the methods and reader skill level of which may be highly variable [15, 33].

Previous studies have concluded that positive coproantigen results have a high level of specificity. One study tested 100 specific-pathogen-free canine fecal samples using the antigen tests for hookworm, ascarid and whipworm, and found a high specificity with all 100 samples testing negative for all three parasites [12, 34]. Additionally, Adolph et al. [14] reported the utilization of antigen detection assays for hookworm, ascarid and whipworm in conjunction with centrifugation increased the sensitivity for the detection of intestinal parasites when using necropsy as the gold standard in a cohort of 97 dogs.

There are several potential limitations to the present study. Comparisons between wellness and non-wellness positivity cannot be construed as a well *versus* sick comparison as the non-wellness category of visits includes procedures such as dental cleanings or surgeries in addition to clinically ill cats. The inclusion of these cases in the non-wellness category may have decreased overall positivity for this population. Although this study encompassed a large geographical population, some areas were underrepresented. Montana (*n* = 0), Mississippi (*n* = 14), North Dakota (*n* = 18), Idaho (*n* = 36), South Dakota (*n* = 70), Wyoming (*n* = 74), Arkansas (*n* = 87), Nevada (*n* = 93), Kentucky (*n* = 97), Nebraska (*n* = 147), Alabama (*n* = 164) and Kansas (*n* = 180) all had fewer than 200 samples meeting inclusion criteria in 2017 and 2018. In addition, the study population consisted of data from a single commercial reference laboratory provider and thus may not reflect the true prevalence of the population. Patient history, physical examination findings, or other diagnostic tests were not available for inclusion in the analysis.

## Conclusions

Intestinal parasitism in companion animals is widely considered more common in juveniles than adults [9, 35]. This study supports the previous findings that younger cats have more intestinal parasites and demonstrates specific trends in intestinal parasitism of domestic cats. Regional and age differences in parasitism help to target at-risk groups that should receive increased fecal testing. Commonly used methods of parasite detection in the USA may underestimate the risk for intestinal parasite infection in cats [1, 9]. This study found that the percentage of samples that tested positive using coproantigen immunoassay testing was consistently higher than the percentage of samples that tested positive using flotation by centrifugation. This trend was consistent across ages, visit types and geographical regions. As a result, adding coproantigen testing to currently recommended centrifugation may lead to increased detection of parasitism in cats.

## Supplementary information

**Additional file 1: Table S1.** Co-infections for hookworms, ascarids, whipworms and *Giardia* in feline centrifugation or coproantigen tests.

**Additional file 2: Table S2.** Ages of cats receiving centrifugation or coproantigen tests on wellness or non-wellness veterinary visits.

**Additional file 3: Figure S1.** Proportion with positive test results for centrifugal flotation and coproantigen by region broken down by United States Census Bureau region, parasite and age category.

**Additional file 4: Table S3.** Positive feline fecal parasite test results by centrifugation or coproantigen.

## Data Availability

All data generated or analyzed during this study are included in this published article and its additional files.

## Abbreviation

CAPC: Companion Animal Parasite Council
