# Supplementary material for: Retrospective analysis of feline intestinal parasites: trends in testing positivity by age, USA geographical region and reason for veterinary visit
Source: Parasit Vectors. 2020 Sep 15;13:473. doi: 10.1186/s13071-020-04319-4 (PMC7493338; doi:10.1186/s13071-020-04319-4)
Supplement: Supplementary file 1 — Additional file 1: Table S1. Co-infections for hookworms, ascarids, whipworms and Giardia in feline centrifugation or coproantigen tests. [file 13071_2020_4319_MOESM1_ESM.docx]

**Additional file 1: Table S1.** Coinfections for hookworm, ascarid, whipworm, and *Giardia* in feline centrifugation or coproantigen tests.

| Species | % |
| --- | --- |
| 4 Coinfections: <0.01% | |
| Hookworm/Ascarid/Whipworm/*Giardia* | <0.01% |
| 3 Coinfections: 0.09% | |
| Ascarid/Whipworm/*Giardia* | <0.01% |
| Hookworm/Ascarid/*Giardia* | 0.07% |
| Hookworm/Ascarid/Whipworm | 0.01% |
| Hookworm/Whipworm/*Giardia* | <0.01% |
| 2 Coinfections: 1.34% | |
| Ascarid/*Giardia* | 0.90% |
| Ascarid/Whipworm | 0.02% |
| Hookworm/Ascarid | 0.33% |
| Hookworm/*Giardia* | 0.07% |
| Hookworm/Whipworm | 0.02% |
| Whipworm/*Giardia* | 0.01% |
| Single Infection: 11.7% | |
| Ascarid | 5.42% |
| *Giardia* | 5.54% |
| Hookworm | 0.63% |
| Whipworm | 0.11% |
