# Supplementary material for: Retrospective analysis of feline intestinal parasites: trends in testing positivity by age, USA geographical region and reason for veterinary visit
Source: Parasit Vectors. 2020 Sep 15;13:473. doi: 10.1186/s13071-020-04319-4 (PMC7493338; doi:10.1186/s13071-020-04319-4)
Supplement: Supplementary file 2 — Additional file 2: Table S2. Ages of cats receiving centrifugation or coproantigen tests on wellness or non-wellness veterinary visits. [file 13071_2020_4319_MOESM2_ESM.docx]

**Additional file 2: Table S2.** Ages of cats receiving centrifugation or coproantigen tests on wellness or non-wellness veterinary visits.

| Visit Type |  | 2-7 mo  N (%) |  | 7-12 mo  N (%) |  | 1-3 yr  N (%) |  | 3-6 yr  N (%) |  | 6-9 yr  N (%) |  | 9-14 yr  N (%) |  | 14+  N (%) |  | Total  N (%) |
| --- | --- | --- | --- | --- | --- | --- | --- | --- | --- | --- | --- | --- | --- | --- | --- | --- |
| Wellness |  | 13680 (28%) |  | 2386 (4.9%) |  | 4035 (8.3%) |  | 8409 (17.2%) |  | 6624 (13.6%) |  | 9209 (18.9%) |  | 4458 (9.1%) |  | 48801 (50.9%) |
| Non-Wellness |  | 6578 (14%) |  | 4398 (9.4%) |  | 4147 (8.8%) |  | 7392 (15.7%) |  | 5889 (12.5%) |  | 10735 (22.8%) |  | 7863 (16.7%) |  | 47002 (49.1%) |
| Total N (%) |  | 20258 (21.1%) |  | 6784 (7.1%) |  | 8182 (8.5%) |  | 15801 (16.5%) |  | 12513 (13.1%) |  | 19944 (20.8%) |  | 12321 (12.9%) |  | 95803 |
|  |  |  |  |  |  |  |  |  |  |  |  |  |  |  |  |  |
