# Supplementary figures and images for: Retrospective analysis of feline intestinal parasites: trends in testing positivity by age, USA geographical region and reason for veterinary visit
Source: Parasit Vectors. 2020 Sep 15;13:473. doi: 10.1186/s13071-020-04319-4 (PMC7493338; doi:10.1186/s13071-020-04319-4)

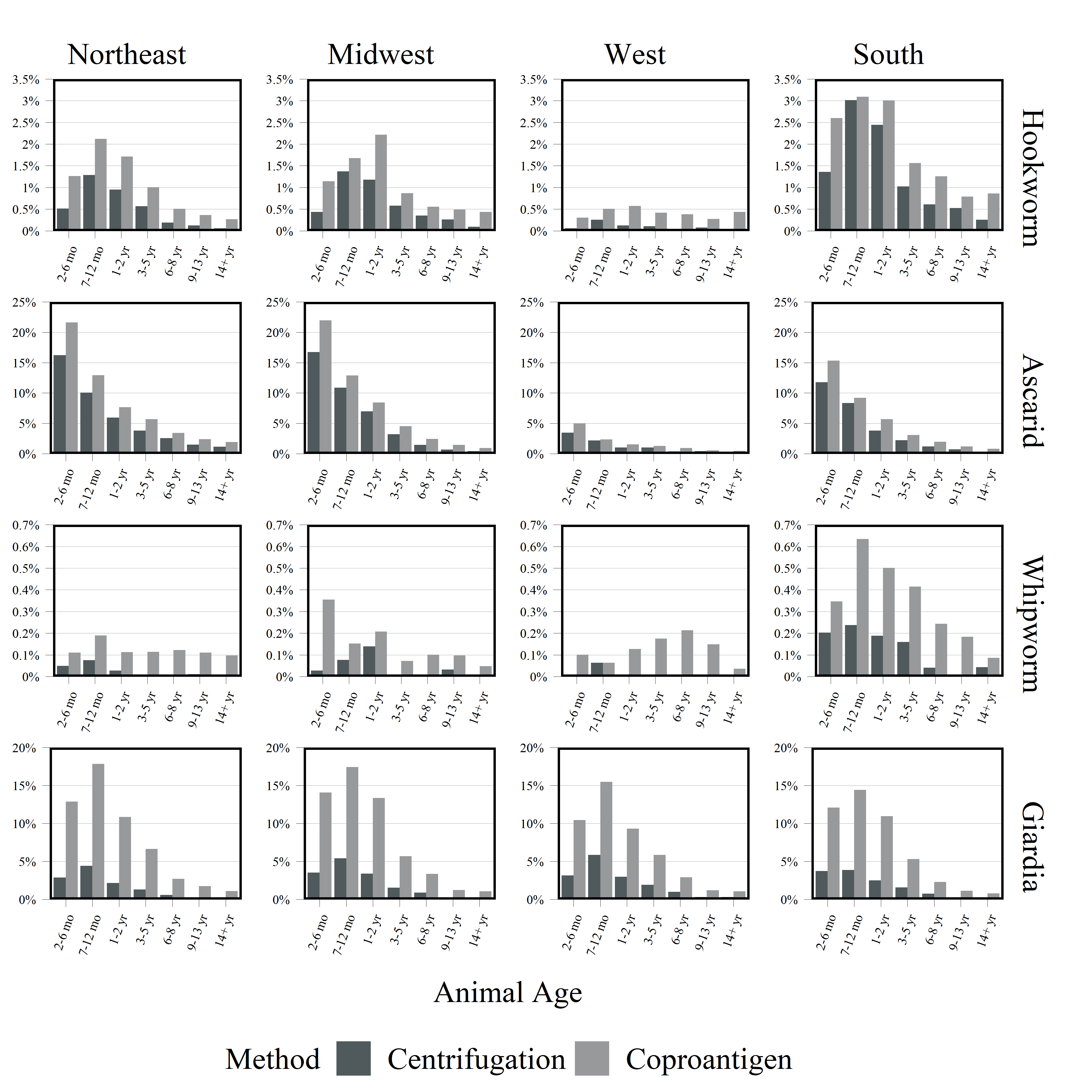

Supplement: Supplementary file 3 — Additional file 3: Figure S1. Proportion with positive test results for centrifugal flotation and coproantigen by region broken down by United States Census Bureau region, parasite and age category. [file 13071_2020_4319_MOESM3_ESM.tiff]
