# Supplementary material for: Retrospective analysis of feline intestinal parasites: trends in testing positivity by age, USA geographical region and reason for veterinary visit
Source: Parasit Vectors. 2020 Sep 15;13:473. doi: 10.1186/s13071-020-04319-4 (PMC7493338; doi:10.1186/s13071-020-04319-4)
Supplement: Supplementary file 4 — Additional file 4: Table S3. Positive feline fecal parasite test results by centrifugation or coproantigen. [file 13071_2020_4319_MOESM4_ESM.docx]

**Additional file 4: Table S3.** Positive feline fecal parasite test results by centrifugation or coproantigen.

| Parasite |  | Positives Both Methods |  | Positives Either Method |  | Positives Coproantigen |  | Positives Centrifugation |
| --- | --- | --- | --- | --- | --- | --- | --- | --- |
| *Giardia* |  | 1536 |  | 6318 |  | 6254 |  | 1600 |
| Hookworm | | 283 |  | 1095 |  | 905 |  | 473 |
| Ascarid |  | 3901 |  | 6467 |  | 5967 |  | 4401 |
| Whipworm | | 16 |  | 173 |  | 155 |  | 34 |
